# A long time radiological follow-up of neuronal intranuclear inclusion disease: Two case reports

**DOI:** 10.1097/MD.0000000000013544

**Published:** 2018-12-10

**Authors:** Linglong Chen, Lin Wu, Shenghong Li, Qin Huang, Jiajun Xiong, Daojun Hong, Xianjun Zeng

**Affiliations:** a Department of Radiology, The First Affiliated Hospital, Nanchang University; b Jiangxi Province Medical Imaging Research Institute; c Department of Neurology, The First Affiliated Hospital, Nanchang University; d Department of Neurology, Peking University People's Hospital, No. 11 Xizhimen South Street,Xicheng District, Beijing, PR China.

**Keywords:** diffusion-weighted imaging, magnetic resonance imaging, neurodegenerative disease, neuronal intranuclear inclusion disease

## Abstract

**Rationale:**

Neuronal intranuclear inclusion disease (NIID) is a rare neurodegenerative disease identified with diffusion-weighted imaging (DWI) high-intensity signal in magnetic resonance imaging (MRI). The disappearance of the abnormal signal is extremely rare.

**Patient concerns:**

We present the 2 cases of patients, both of them were suffering from heterogeneous symptoms. We followed up one of them for 7 years with MRI, the other accepted comprehensive MRI inspections.

**Diagnoses:**

DWI high-intensity signal were observed along the corticomedullary junction in MRI plan scan of heads of 2 patients. For patient 1, the hyperintensities in DWI and fluid-attenuated inversion recovery (FLAIR) images in the occipital lobe disappeared 5 years after onset. Based on the biopsy, patient 1 and 2 were diagnosed as NIID.

**Interventions:**

There have not effective medication and prevention for NIID. Patient 1 and 2 received symptomatic treatment.

**Outcomes:**

Up until now, the patients are alive but the disease is progressing.

**Lessons:**

DWI high-intensity signal is a strong clue for the diagnosis of NIID, but the rare case of the disappearance of it may lead to misdiagnosis.

## 1 Introduction

Neuronal intranuclear inclusion disease (NIID) is a rare neurodegenerative disease characterized by hyaline intranuclear inclusions which could be stained with anti-ubiquitin and eosin. The first case of NIID was reported by Lindenberg in 1968,^[1]^ and the skin biopsy as a regarded method was used for the first time in 2011 by Sone.^[1]^ For the variety of clinical features and pathological findings, NIID was considered as a heterogeneous disease.^[2,3]^ As an important auxiliary inspecting method, the high-intensity signal in the corticomedullary junction in diffusion-weighted imaging (DWI) images is characteristic clues for diagnosis, yet there are few long-term radiological follow-ups of NIID. Herein, we report 2 cases of NIID confirmed in our hospital.

## 2 Case report

Case 1: A 58-year-old man visited our hospital for paroxysm, progressive declines in memory and cognition and slow response for the first time in 2011. The patient had a long course of illness and showed a progressive tendency, with refractory hypoglycemia and no history of hypertension. The onset of hypomnesis, dysuria, and dry stools began in 2009. In 2010, the symptoms of dry skin, no sweat, paroxysmal fever, fatigue, and unstable walking appeared. During this period, the patient had a plantar sensation and paresthesia and was misdiagnosed as “viral encephalitis” and “immune encephalitis”. There was not any similar history in his family.

**Figure 1:**
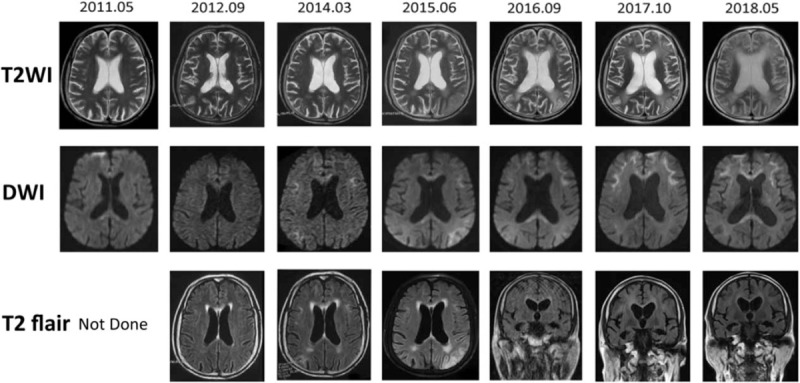
Seven years follow-up of patient 1. The DWI high-intensity signals were observed in the frontal lobes at first and spreading backward along corticomedullary junction. In the image of 5 years after onset, the abnormal DWI signal disappeared. DWI = diffusion-weighted imaging.

Several times of magnetic resonance imaging (MRI) plan scan (Fig. 1) were performed in our hospital, revealed the changing process of the disease. There was no obvious abnormal signal or lesions in the early stage in 2011 and 2012. The subcortical linear hyperintensities in DWI appeared in 2014, predominantly in the frontal lobes, secondly in the occipital lobe, corresponding with hyperintensities in fluid-attenuated inversion recovery (FLAIR) images. The abnormalities in the frontal lobes spread along the corticomedullary junction as the disease progression but did not expand into the deep white matter even in the latest MRI scan. On the contrast, the hyperintensities in DWI and FLAIR images in the occipital lobe disappeared 5 years after onset, together with the T2 weighted images hyperintensities.

Finally, the skin biopsy of patient 1 was performed and the tissue was taken from the 10 cm superior of the lateral malleolus and sectioned in 6 μm thickness. The diagnose of NIID is more obvious by finding anti-ubiquitin staining in nuclear (Fig. 3A). The patient is receiving symptomatic treatment in our hospital and the symptoms are relieved.

Case 2: A 60-year-old female patients visited our hospital for symptoms of walking instability, dizziness, headache, poetic language, blurred vision, bucking, difficulty swallowing lasting for 5 months. After receiving symptomatic treatment in the local hospital, these symptoms got worse. The specialty check-up of our hospital showed that the patient was conscious, with a slow gait, vague speech, degenerated muscle strength, decreased muscle tension and normal tendon reflex. And the laboratory examinations were all within the normal limit. There was no similar history of her family.

**Figure 2:**
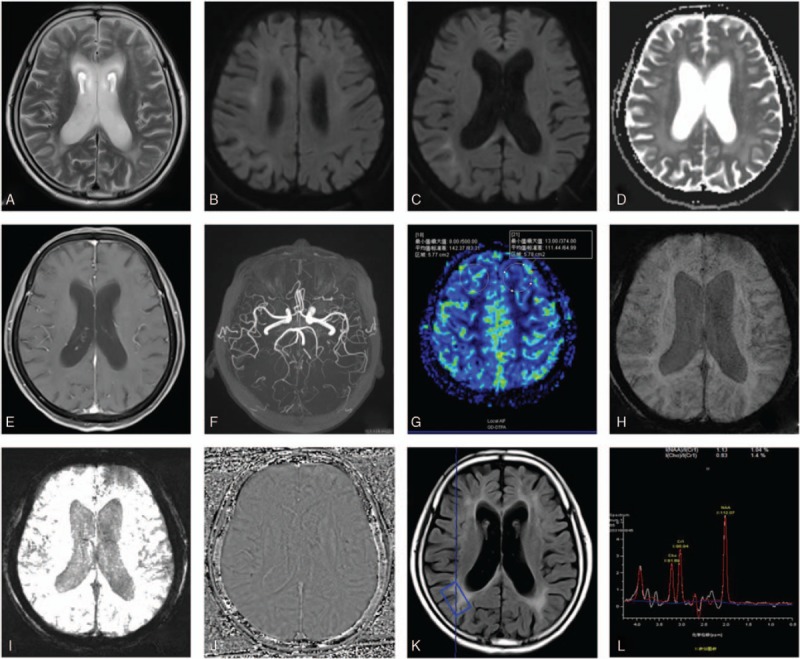
The MRI inspections of the second patient. The DWI (B–C), FLAIR (K) and T2WI (A) high-intensity signals were observed in the frontal lobe and the occipital lobe, and meanwhile, there were not obviously abnormality in the contrast-enhanced MRI (E), the magnetic resonance angiography (F), susceptibility weighted imaging (H–J), the MRS images (L) and cerebral blood flow images (G). DWI = diffusion-weighted imaging, FLAIR = fluid-attenuated inversion recovery, MRI = magnetic resonance imaging, MRS = magnetic resonance spectroscopy.

Comprehensive MRI inspections were done at our hospital. MR images showed a mild degree of cerebral atrophy, with a slight widening of the hemispheric sulci. High-intensity lines along with corticomedullary junction appeared symmetrically in DWI images (Fig. 2 B–C) together with the T2 weighted images and FLAIR hyperintensities (Fig. 2A). There was no abnormality in gadolinium-enhanced images or 3D time-of-flight images or the series of susceptibility weighted imaging. The cerebral blood flow of the left frontal lobes is slightly decreased compared with the right part. The peak value of Cho, NAA or Cr in MR Spectroscopy had no obvious abnormity.

**Figure 3:**
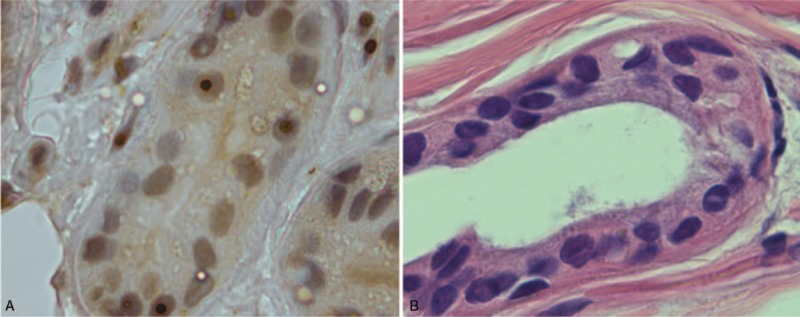
Histological features of the case. Intranuclear inclusions were found stained with anti-ubiquitin (A), and eosin (B).

The skin biopsy of patient 2 was carried out. Hematoxylin-eosin staining showed intranuclear inclusions in the specimens of the second patient (Fig. 3B). The diagnose is NIID. The patient is receiving symptomatic treatment in our hospital and the symptoms are relieved.

The patients provided written informed consent for reporting the case details. In this case, the patient accepted the standard, proven diagnosis, and therapy in the Department of Neurology, so ethical approval was not necessary.

## 3 Discussion

NIID is considered to be a heterogeneous neurodegenerative disease for various clinical symptoms, and dementia is the most common.^[4,5]^ Other symptoms such as autonomic impairment, muscle weakness, disturbance of consciousness, abnormal behavior, sensory disturbance or the so-called frontal signs, also appeared as the disease progressed. The onset age of NIID varies from infant to late middle age, and the patients can be divided into 3 subgroups: infantile, juvenile, and adult. The variety of symptoms and onset age may present a difficult challenge for diagnosis. In all NIID patients, hyaline intranuclear inclusions could be discovered in the central and peripheral nervous systems, or easily detected in the visceral organs such as sweat gland cells by skin biopsy.^[6]^ Since the skin biopsy was reported to be useful for diagnosis of NIID, the number of researches combined pathology with MRI is increasing.^[6]^ Currently, there is no effective treatment of NIID, and patients receive curative therapy.

Our case is unique because we reported 2 sporadic cases of NIID patients with long time follow-up and comprehensive MRI inspections, and one of them showed the disappearance of DWI high intensity signal after 5 years of onset. In MRI, NIID showed leukoencephalopathy with the high intensity signal in DWI and FLAIR. Previous researches have indicated that the continuous linear DWI high-intensity signal which rarely extended into the deep white matter is a characteristic feature for diagnosis.^[7]^ Abnormal hyperintense signal firstly appeared in the frontal lobe, then spread to the parietal and occipital lobe. The signal distributed symmetrically along the corticomedullary junction, related with spongiotic changes in white matter proximal to the U-fibers. And the high intensity in FLAIR, though not typical enough, correlated with diffuse myelin pallor without spongiosis.^[8]^ The disappearance of abnormal signal in DWI is rarely reported,^[9]^ reversed to the common idea that this signal would not disappear once appeared. One of the possible reasons is the absorption of edema. Another similar phenomenon in MRI was reported at the late stage of Creutzfeldt-Jakob disease.^[10]^ Subsequent neuronal loss and gliosis may reduce the increased signal in DWI, and also account for irreversible brains shrink.

The clue of a hyperintense signal in DWI also points to other neurologic diseases such as status epilepticus, Creutzfeldt-Jakob disease, mitochondrial encephalomyopathy. However, there are not obviously abnormal in MR Spectroscopy, cerebral blood flow or 3D time-of-flight images, indicating that our cases do not belong to a kind of vasogenic or metabolic disease. Comprehensive MRI inspections help identify NIID with other magnetic resonance sequences.

In conclusion, DWI high intensity signal is a strong clue for the diagnosis of NIID, but the rare case of the disappearance of it may lead to misdiagnosis. It is necessary to combine with a series of comprehensive inspections and skin biopsy to make a definite diagnosis.

## Author contributions

**Conceptualization:** Linglong Chen and Shenghong Li.

**Investigation:** Linglong Chen and Daojun Hong.

**Resources:** Shenghong Li, Qin Huang, Jiajun Xiong, and Daojun Hong.

**Writing – original draft:** Linglong Chen.

**Writing – review & editing:** Lin Wu and Xianjun Zeng.
